# Automated annotation error detection and correction for manual two-dimensional cinematic magnetic resonance imaging segmentations using segment anything 2

**DOI:** 10.1016/j.phro.2026.101019

**Published:** 2026-06-13

**Authors:** Pia A.W. Görts, Mariska de Smet, Shyama U. Tetar, Heike M.U. Peulen, Miguel A. Palacios, Coen Hurkmans, Marcel Breeuwer, Rob H.N. Tijssen

**Affiliations:** a Department of Radiation Oncology, Catharina Hospital Eindhoven, Eindhoven, the Netherlands; b Department of Biomedical Engineering, Technical University Eindhoven, Eindhoven, the Netherlands; c Department of Radiation Oncology, Amsterdam UMC, location VUmc, Amsterdam, the Netherlands; d Department of Electrical Engineering, Technical University Eindhoven, Eindhoven, the Netherlands; e Department of Applied Physics and Science Education, Technical University Eindhoven, Eindhoven, the Netherlands

**Keywords:** MRI-guided radiotherapy, Medical image segmentation, Deep learning, Dataset cleaning

## Abstract

Performance of deep learning-based models for tumour tracking in magnetic resonance-guided radiotherapy hinges on high-quality labelled data. Medical images are prone to annotation errors, making data cleaning essential. We propose an automatic data cleaning tool based on the foundation model Segment Anything 2, which incorporates temporal information from cinematic magnetic resonance imaging to detect annotation errors and generate corrected contours, minimising manual effort involved in data cleaning. Expert validation by two radiation oncologists showed a preference for corrected contours over the original manual contours. Corrected contours (DSC 0.95 ± 0.01) surpassed interobserver variability (0.88 ± 0.02) on a dataset annotated by five observers.

## Highlights

• A novel data cleaning tool for cinematic magnetic resonance imaging segmentations.
• Uses a foundation model to incorporate temporal context to detect annotation errors.
• Flags and corrects erroneous frames, identifying approximately 6% as outliers.
• Corrected contours were favoured over the original manual segmentations.
• Corrected contours showed good agreement (DSC = 0.95 ± 0.01) with STAPLE contours.

## Graphical abstract

**Figure:**
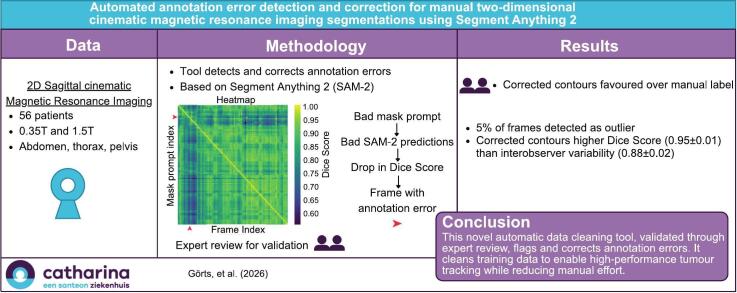

## 1 Introduction

Tumour motion during radiation therapy (RT) can affect accurate dose delivery. The Magnetic Resonance Linear Accelerator (MR-Linac) mitigates this through beam gating by only turning the beam on when the target is within the tracking boundary. Gating requires target tracking using cinematic magnetic resonance imaging (cine-MRI) frames, which is clinically done by deformable image registration (DIR) [1]. However, deep learning (DL) models may offer a faster and potentially more accurate alternative [2].

Supervised DL performance hinges on high-quality labelled training data. Ideally, annotations are created by multiple experts to minimise interobserver variability. Manual delineation is highly time-consuming and therefore often performed by one observer, increasing the likelihood of labelling errors that can degrade model performance. Systematic dataset cleaning is therefore essential [3]. Cleaning can be done manually or automatically using dedicated tools.

Most existing data cleaning tools target classification rather than segmentation [4]. Cleanlab, proposed by Lad et al., uses probability outputs from a trained segmentation model to correct annotation errors, but has not been applied to medical data [5]. Jin et al. introduced a probability-based pixel-level correction method for breast ultrasound images [6]. Liu et al. proposed Adaptive Early Learning Correction (ADELE) to correct noisy computed tomography labels during training [7]. However, these approaches are designed for static images, meaning that temporal information is lost when applied to cine-MRI.

The aim of this study was to develop a dataset cleaning tool that identified annotation errors in cine-MRI segmentations and provided alternative corrected contours. We proposed a method based on the foundation model Segment Anything 2 (SAM-2) [8], which is highly generalisable and has been shown to be effective on cine-MRI data [9]. Unlike earlier approaches, the proposed tool incorporated temporal information across images and was validated through expert review.

## 2 Materials and methods

### 2.1 Data

This study used labelled data from the TrackRAD2025 challenge [2], comprising sagittal two-dimensional (2D) breath-hold cine-MRI time-series from 108 patients treated on 1.5 T Elekta Unity (Elekta AB, Stockholm, Sweden; 56 patients) or 0.35 T MRIdian (ViewRay Inc., Oakwood Village, OH, USA; 52 patients) MR-Linac systems across five institutions. This research was conducted on anonymised patient data and was approved for a waiver under the Dutch medical research law. Targets included the Gross Tumour Volume (GTV) or surrogate structures (e.g., whole liver) across the thorax, abdomen, and pelvis. Time-series contained 44–239 frames due to variable frame rates (1.62–8 Hz) and treatment durations. Training and test sets of the challenge were used. From the test set, only 0.35 T cases with five independent annotations were retained to obtain a high-quality subset. One or two observers annotated the training set. For multi-annotator cases, a single ground truth was generated using the simultaneous truth and performance level estimation (STAPLE) algorithm [10]. To make the datasets more homogeneous, targets larger than 25 cm^2^ were excluded to exclude surrogate structures such as the liver, resulting in 42 training patients and 14 testing patients (datasets D1 and D2, respectively). For one case with out-of-plane motion, only frames containing a visible target were retained. Further dataset details are provided in the TrackRAD2025 dataset paper [2]. All processing and analyses were performed in Python 3.12.

### 2.2 Data cleaning method

**Fig. 1:**
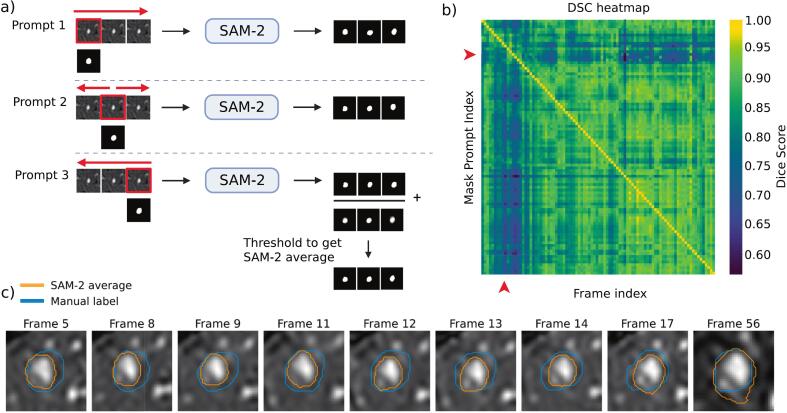
Workflow for the data cleaning method. (a) The red boxes depict the frame used as a mask prompt. Segmentation was performed forward and backward to ensure that the prompt always served as the starting point. (b) DSC heatmap for a patient from dataset D1. The red arrows indicate the rows and columns with a low DSC score, which may contain annotation errors. (c) Outlier frames flagged from the DSC heatmap, together with contours of the manual label and SAM-2 average contour. Created in BioRender. Görts, P. (2026) https://BioRender.com/cbu4ypz (For interpretation of the references to colour in this figure legend, the reader is referred to the web version of this article.)

SAM-2 [8] was used to segment entire cine sequences using a single mask prompt. The cleaning tool leveraged the dependence of SAM-2's prediction accuracy on prompt quality, which is tied to the manual label. As illustrated in Fig. 1, each of the N frames was used as a mask prompt to segment all N frames, yielding N x N SAM-2 predictions per patient. A heatmap of Dice Similarity Coefficient (DSC) scores between SAM-2 outputs and manual labels was generated. In each row, the prompt is fixed, and the segmented frame varies, whereas in each column, the prompt varies and the segmented frame is fixed. Row and column DSC averages were computed, and outliers were flagged using Tukey's IQR rule (Q_1_–1.5 · IQR) [11], indicating potential labelling errors in the manual labels.

A drop in DSC may be due to an inaccurate mask prompt, producing poor SAM-2 outputs throughout the entire sequence, thereby correctly flagging outlier frames. False positives may be caused by SAM-2 failing on specific frames due to artefacts or contrast differences, regardless of the prompt used.

### 2.3 SAM-2 average contour quality

Additional to outlier frame detection, a SAM-2 average contour was generated for each frame by averaging the N predictions from different mask prompts, thereby removing the prompt bias. After outlier detection, users were prompted to inspect the outlier frames and remove or correct the manual label or replace it with the SAM-2 average contour, which is useful for models requiring frame continuity. Outlier detection and SAM-2 average computation were performed on datasets D1 and D2.

To assess if the SAM-2 average contour offers a good alternative for the outlier labels, DSC scores, and 95th percentile Hausdorff distance (HD95) between the SAM-2 average and STAPLE contours of dataset D2 were compared to the interobserver variability. Temporal consistency was assessed using the mean absolute deviation (MAD) of SAM-2 average and manual contours of datasets D1 and D2. The MAD, defined in Eq. (1), computes the average difference of each frame's area $x_{i}$ from the mean area $\bar{\mu }$ across N (frames)

(1) $$\mathrm{MAD}=\frac{1}{N}\sum_{i=1}^{N}|x_{i}-\bar{\mu }|$$

### 2.4 Expert review

An expert review was conducted to assess SAM-2 average contour quality. Two radiation oncologists performed a blinded review of 30 consecutive frames from 10 randomly selected patients from dataset D1 (all 0.35 T). For each frame, observers were shown the SAM-2 default contour (using frame 0 as prompt), the SAM-2 average contour, and the manual label. From the three blinded contours, experts indicated which segmentation(s) they deemed correct, with the option to select one, multiple, or none of the contours. The frequency with which a contour was selected as correct was analysed for both observers.

## 3 Results

The outlier detection tool ran on an NVIDIA RTX A6000 GPU for 0.4 · N^2^ seconds for a sequence of N frames. In dataset D1, detected outliers ranged from 0.0% to 14.0% of the frames (mean ± SD: 5.7 ± 3.6%), and in dataset D2, from 1.0% to 15.0% (6.1 ± 4.1%). The DSC thresholds used to identify outliers were 0.88 ± 0.05 (rows) and 0.86 ± 0.07 (columns) for dataset D1, and 0.93 ± 0.02 (rows) and 0.92 ± 0.02 (columns) for dataset D2.

For dataset D2, the SAM-2 average contours achieved a DSC of 0.95 ± 0.01 and an HD95 of 2.27 ± 0.43 mm relative to STAPLE labels. Pairwise interobserver variability yielded a DSC of 0.88 ± 0.02 and an HD95 of 4.60 ± 0.50 mm.

Temporal consistency analysis showed that, in dataset D1, the MAD was 36 ± 46 mm^2^ for the SAM-2 average and 50 ± 50 mm^2^ for manual labels (Wilcoxon *p* < 0.001). In dataset D2, MAD values were 43 ± 26 mm^2^ and 41 ± 32 mm^2^ for SAM-2 average and manual labels, respectively (*p* = 0.39).

**Fig. 2:**
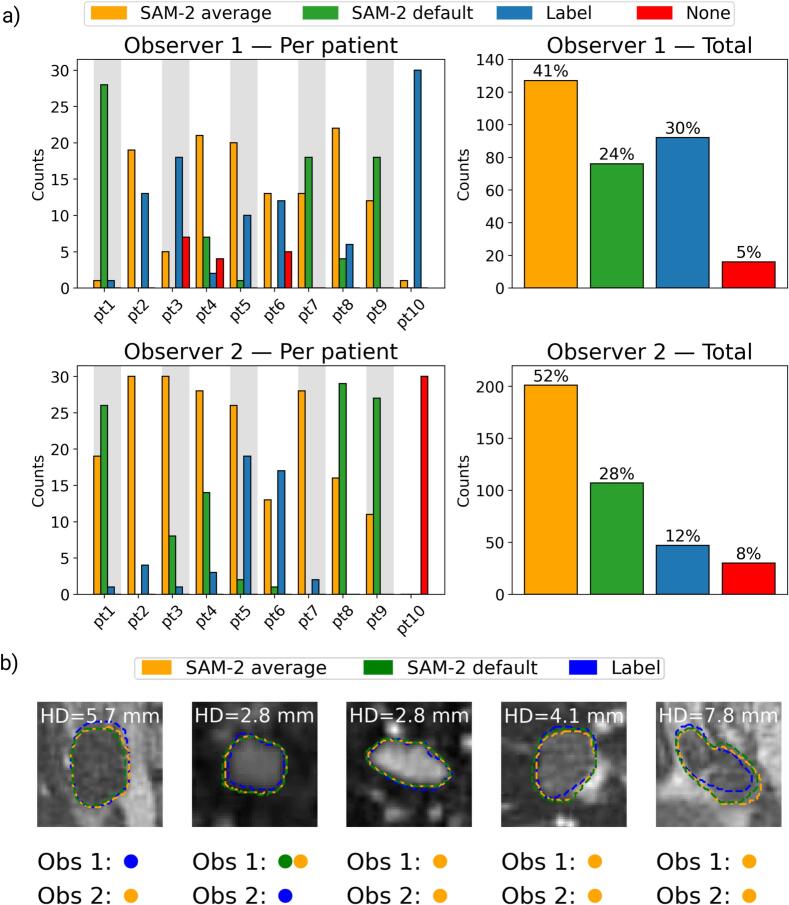
(a) The left subplots show a patient-level result of the frequency with which each contour was chosen as correct segmentation for both observers. Patients are randomly selected from dataset D1 and all acquired at 0.35 T. The figure shows a high per-patient and interobserver variability. The right subplots show the group average results for all patients. The SAM-2 average contour was chosen most frequently by both observers. (b) Examples of outlier frames that were detected by the outlier detection tool among the 300 frames that were reviewed. The generated contours, as offered to the experts, are also shown, along with the ‘correct contours’ identified by both observers. The maximum Hausdorff distance (HD) between the three contours is shown as a scale indication.

Fig. 2a shows that both observers most frequently selected SAM-2 average contours as correct. Among the 300 reviewed frames, the tool flagged nine outlier frames. In eight of these cases, both observers agreed with the tool and deemed the manual label incorrect. Fig. 2b shows example outlier frames and contours, along with the experts' choices. The mean DSC between the label and SAM-2 default, the label and SAM-2 average, and SAM-2 default and SAM-2 average were 0.90 ± 0.05, 0.92 ± 0.05, and 0.94 ± 0.03, respectively.

## 4 Discussion

This study succesfully developed an automatic data cleaning tool to obtain high-quality labels necessary for training accurate DL-based tracking models. We validated a SAM-2-based tool that flags frames with annotation errors and offers alternative contours. Results showed that this approach produces reliable corrected contours, improves temporal consistency, and effectively flags outlier frames. By reducing the manual effort required for dataset cleaning, this approach facilitated the creation of high-quality labelled datasets, which are essential for the development of accurate deep learning based tracking models.

Expert review supported the SAM-2 average contour as a potential alternative to the manual label, as it was chosen most frequently by experts. High DSC scores across SAM-2 default, SAM-2 average, and manual contours indicate strong similarity, making it difficult to identify correct tumour segmentations, a point that was agreed upon by the experts. This was reflected in large per-patient variability and limited interobserver agreement. Patient 10 illustrates this clearly: Observer 2 selected ‘no preference’ for all frames, as the 2D sagittal image alone was insufficient for decision-making and a 3D MRI would have offered more anatomical context.

SAM-2 average contour quality was further supported by achieving a higher DSC and lower HD95 than the interobserver variability when compared with the STAPLE labels of dataset D2, demonstrating higher consensus with STAPLE labels than among the five observers. A group-based analysis of the anatomical sites showed a maximum difference of 0.4% in detected outliers and no correlation between motion and the number of detected outliers, suggesting no variation in performance levels across anatomical sites.

In dataset D1, the SAM-2 average MAD was significantly lower than that of the manual labels, while in dataset D2 both were similar, showing that SAM-2 achieves temporal consistency comparable to multi-observer annotations.

Outliers were identified using a Tukey IQR-based threshold, which flags about 0.7% of the frames as outliers for normally distributed data, with higher rates expected for skewed data [12]. Thresholds of dataset D2 were 5% (rows) and 7% (columns) higher than for dataset D1, reflecting smaller differences between SAM-2 outputs and manual labels in dataset D2, as expected given its higher-quality annotations. Thresholds can be adjusted in future work depending on the desired sensitivity of outlier detection.

Lad et al. [5] showed high precision and recall of Cleanlab on natural images, but was not evaluated on medical data. Liu et al. [7] and Jin et al. [6] demonstrated improved performance when training with their proposed cleaning strategies. However, neither included expert review of corrected labels, limiting direct comparison with our results. These methods process frames independently, causing temporal information loss in cine images. In contrast, our method leverages SAM-2's memory bank to incorporate temporal information to maintain temporal consistency in corrected labels. Future work should investigate the effect of using our method for training data cleaning on model performance. Although our method is more computationally expensive than the other three methods, it still significantly reduces manual labour. Lombardo et al. reported that manually segmenting just eight 2D cine-MRI frames took three minutes, demonstrating how quickly annotation time increases when scaled up to larger datasets required for DL models [13].

A disadvantage of the IQR rule is that it may flag high-quality labels as outliers when SAM-2 outputs and labels are similar (i.e., time-series with good label quality). This can be avoided by manually inspecting outlier frames. Although still requiring some manual work, it is significantly faster than manually reviewing all frames.

Since the tool's runtime scales quadratically with time-series length, processing long time-series can become computationally expensive. To reduce computation time, efficiency improvements such as splitting the series into multiple shorter series could be explored.

Evaluation was limited to patients for whom the GTV was tracked, as surrogate tracking data were scarce within the TrackRAD2025 dataset. In addition, analysis was restricted to cine sequences in which the target remained within the imaging plane. If sequences with out-of-plane motion are to be included, frames without the target could be excluded, with analysis resuming upon target reappearance. Given the robustness of SAM-2 to non-continuous image sequences, this strategy is unlikely to adversely affect performance.

We proposed a novel data cleaning method that leverages the foundation model SAM-2 and incorporates temporal information from cine-MRI. Corrected contours and the STAPLE labels showed higher consensus than that among the five observers contributing to the STAPLE labels. The MAD also confirmed an increased temporal consistency for the SAM-2 average contours. Furthermore, experts favoured the SAM-2 average contour over the manual label for most reviewed frames. After outlier detection, users can review the frames and choose to delete, keep, or replace the manual labels with corrected contours. Corrected contours could improve consistency and reduce temporal variation in DL datasets. Although computationally expensive, the method significantly reduces manual labour to obtain more geometrically consistent training datasets and works for any dataset with temporally correlated frames. High-quality data is essential for the development of high-accuracy DL-based tumour tracking algorithms.

## CRediT authorship contribution statement

**Pia A.W. Görts:** Writing – original draft, Visualization, Software, Methodology, Investigation, Formal analysis, Conceptualization. **Mariska de Smet:** Writing – review & editing, Formal analysis, Data curation. **Shyama U. Tetar:** Writing – review & editing, Formal analysis, Data curation. **Heike M.U. Peulen:** Writing – review & editing, Formal analysis, Data curation. **Miguel A. Palacios:** Writing – review & editing, Data curation. **Coen Hurkmans:** Writing – review & editing, Supervision, Methodology, Conceptualization. **Marcel Breeuwer:** Writing – review & editing, Supervision, Methodology, Conceptualization. **Rob H.N. Tijssen:** Writing – review & editing, Supervision, Methodology, Data curation, Conceptualization.

## Declaration of generative AI and AI-assisted technologies in the writing process

During the preparation of this work, the authors used Copilot (Microsoft) to enhance readability and proofread the English text. After using this tool, the authors reviewed and edited the content as needed and take full responsibility for the content of the published article.

## Declaration of competing interest

The authors declare that they have no known competing financial interests or personal relationships that could have appeared to influence the work reported in this paper.
